# Common Temporization Techniques Practiced in Saudi Arabia and Stability of Temporary Restoration

**DOI:** 10.1155/2021/4965500

**Published:** 2021-11-09

**Authors:** Fahda N. Algahtani, Reem M. Barakat, Bashayer S. Helaby, Manar A. Alhefdhi, Munirah S. Binshabaib, Lama A. Alrasheed, Mohammed H. Mashyakhy

**Affiliations:** ^1^Clinical Dental Sciences Department, College of Dentistry, Princess Nourah Bint Abdulrahman University, Riyadh, Saudi Arabia; ^2^Preventive Dental Sciences Department, College of Dentistry, Princess Nourah Bint Abdulrahman University, Riyadh, Saudi Arabia; ^3^Health Science Research Center, Princess Nourah Bint Abdulrahman University, Riyadh, Saudi Arabia; ^4^Restorative Dental Science Department, College of Dentistry, Jazan University, Jizan, Saudi Arabia

## Abstract

**Introduction:**

Coronal leakage is detrimental to the long-term success of root canal treatment (RCT). While much emphasis is being placed on the quality of the final restoration, little attention is given to the temporary restoration placed in between root canal treatment appointments. The aim of this study was to survey temporization techniques practiced in Saudi Arabia and the frequency of observing temporary material breakdown or complete loss.

**Materials and Methods:**

An online questionnaire was distributed among general dentists, dental specialists, and clinical trainees in undergraduate and postgraduate dental programs. The sample size was estimated at 370 participants. Data were analyzed using descriptive statistics and chi-square tests.

**Results:**

The total number of participants who met the inclusion criteria was 525. The majority of them (94.6%) were practicing two-visit RCT. The most common temporization materials were Cavit (50.3%) followed by glass ionomer cement (32%). The majority (72.6%) of participants claimed they allow a thickness of 2-3 mm for temporary restorations. Many participants (60.4%) used a spacer material during temporization, and the cotton pellet was the most common spacer material. Temporary restoration breakdown or complete loss was a common observation. Although the duration between the two RCT visits was 2 weeks or less for 83.6% of participants, only 19.6% of participants claimed that they rarely observed temporization breakdown.

**Conclusion:**

Two-visit RCT is commonly practiced in Saudi Arabia, and endodontists performed significantly more single-visit procedures. Temporization practices may lack uniformity; however, clinicians were more likely to use calcium sulfate-derived material for two weeks or less. They allow for 2-3 mm thickness restoration and use a cotton pellet as a spacer. According to their clinical observation, temporary material breakdown or complete loss was frequent. This mandates further attention in research and education.

## 1. Introduction

Root canal treatment (RCT) is a biologically driven procedure that aims to maintain and restore the health of periapical tissue [1–3]. Therefore, bacterial removal and the prevention of recontamination of the root canal system are mandatory for the success of endodontic therapy [1–4]. Coronal leakage has been cited as a major reason for root canal failure [5]. While much emphasis is placed on the quality of the final restoration, little attention was given to temporary restoration placed in between RCT appointments [6, 7].

Temporary restorations are used in restorative dentistry to temporarily restore prepared teeth before the placement of the indirect restoration [1]. The quality of the temporary restorative material is a key indicator for the success of the final restoration [1]. Alternatively, temporary materials such as intermediate restorative material IRM can be used to temporize deep cavities in stepwise excavation or to relieve sensitivity to composite restoration [8]. However, their main use in endodontics is to cover the orifice of the root canal system in between two dental visits or after completing the procedure [8]. Lack of satisfactory temporary restoration during endodontic therapy was the second most contributing factor for intraoperative pain [9]. After the initiation of the root canal procedure, the lumen of the canal is enlarged, which increases the risk of bacterial and food ingress from oral fluid in the absence of adequate restorative sealing [10, 11].

Endodontic emergency treatment followed by temporization was a clinical route of intervention for symptomatic cases during the COVID-19 pandemic [12, 13]. However, fractured temporary restoration was the first cause of negative outcomes in these cases followed by an endodontic flare-up [14]. The type of temporary restoration material is an important factor to ensure the longevity of temporization [15]. A satisfactory temporary restorative material needs to have adequate strength and hardness to resist mechanical load and wear when used in small quantities. In addition, it should adhere to cavity or surrounding restorative margins, should have low solubility and quick setting in the oral environment, is dimensionally stable, and is easy to insert and remove [16].

The technique used for temporization is also important for preserving the quality of temporary restoration for an extended period [16]. For example, the placement of cotton pellet beneath the temporary filling was regarded as a common practice because it facilitates the removal of temporary restoration and locating root canals orifices [17]. This spacer material, however, can act as a cushion that permits material deterioration during mastication and allows for fiber entrapment at restorative margins which compromises the seal of temporization [18, 19]. On the contrary, the placement of a band in restoratively compromised cases in which the tooth is missing the lingual or buccal wall was emphasized to reduce the incidence of breakdown or complete loss of interim and temporary restoration under masticatory forces [16, 20].

The primary aim of this study was to survey the common temporization techniques practiced in Saudi Arabia. The frequency of the clinical observation of temporary material breakdown and/or complete loss was also examined as a secondary aim.

## 2. Materials and Methods

This is a cross-sectional observational study that was exempted from ethical approval by the Institutional Review Board at Princess Nourah Bint Abdulrahman University (PNU) (No. H-01-R-059).

The online survey was distributed from 15^th^ May 2020 to 31^st^ August 2020, targeting dental professionals practicing in Saudi Arabia: general dentists, dental specialists, dental students, and interns. According to the Saudi Commission of Health Specialties (SCFHS), the number of active registered dental practitioners was estimated to be 17201 general dentists and 1507 restorative and endodontic specialists including postgraduate residents. The number of dental students and interns released by the Ministry of Education in Saudi Arabia was 7414.

The sample size (*n*) was calculated according to the following formula:

(1) $$\begin{array}{c} n=\frac{z^{2}\times p\left(1-p\right)/e^{2}}{1+\left(z^{2}\times p\left(1-p\right)/{\text{Ne}}^{2}\right)}, \end{array}$$

where *z* = 1.96 for a confidence level (*α*) of 95%, *p* = proportion (expressed as a decimal), *N* = population size, and *e* = margin of error [21]. The desirable sample size was 379 participants.

The questionnaire was designed to start with the consent of approval for participation, two demographic questions, eight five-point Likert format questions, and five multiple-choice questions that gather information related to the objectives of the study (Table 1). The questionnaire was tested for readability and piloted in a convenient sample of expert members of the dental assessment unit inside the dental school of PNU, one experienced biostatistician, endodontist, and general dentists. Modifications in the questionnaire were done accordingly.

The survey was then emailed to actively registered dental professionals in the SCFHS and the deans of dental schools in Saudi Arabia who were asked to e-mail the survey to concerned dental students, interns, and postgraduate residents. The survey was also disseminated online in populated Saudi dental social media Twitter accounts that have over 10000 followers. Data were analyzed using the JMP14 software (SAS Institute, Cary, North Carolina, United States of America). Descriptive statistics were obtained for all variables examined in the study. Chi-square tests for proportions were used to examine the presence of significant association between demographic variables and practiced techniques. The alpha level was set at 0.05, and *p* values that were less than the alpha level 0.05 were considered significant.

## 3. Results

**Figure 1:**
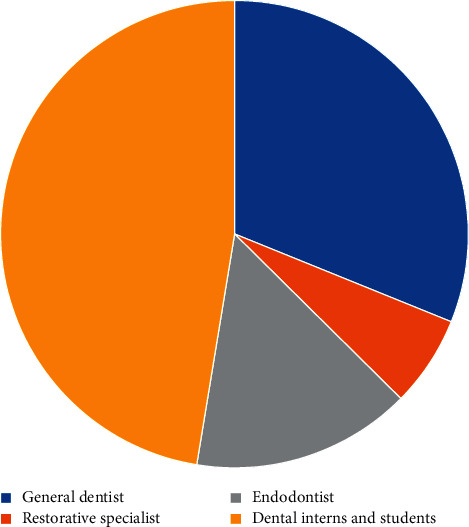
Distribution of participants based on their last degree of qualification.

607 participants agreed to participate in the study. The total number of participants that met the inclusion criteria and answered all requested questions was 525. The number of participants who were practicing dentistry in the government sector was 319 (60.8%) compared to 206 (39.2%) practicing in the private sector. 47.4% of the participants were interns or dental students followed by general dentists (31.1%), endodontists (15.2%), and restorative specialists (6.3%) as shown in Figure 1.

**Figure 2:**
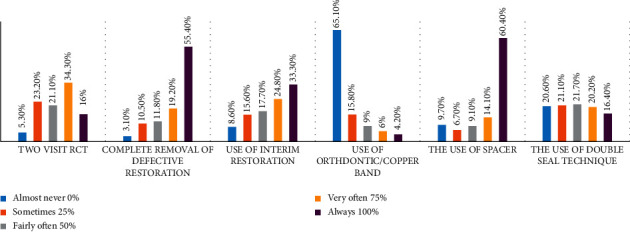
The frequency of two-visit RCT and the common practices of temporizing the root canal system in between the dental visits.

The frequency of doing two-visit RCT and the common practices in temporization in between RCT visits are shown in Figure 2. Only 5.3% of participants completed the RCT in one visit, while the rest of the participants perform two-visit RCT in variable frequencies.

More than half of the participants (55.4%) always removed defective restorations before initiating RCT, while only 3.1% maintained such restorations during RCT.

The participants used an interim restoration to support the new temporary restoration except for 8.6% of the participants who never used this technique. Orthodontic or copper bands were rarely used to support the temporary restoration for 65.1% of the participants. The insertion of spacer such as cotton pellet or foam pellet before temporization was a common practice, in which 60.4% of the participants always use them. Moreover, 20.6% of the participants rarely use the double seal technique for temporization, while 16.4% of the participants always use this technique.

The duration between the two RCT visits was 2 weeks or less for 83.6% of participants and less than one month for 12.8% of the participants. The participants were less likely to spend more than one month (2.3%) or three months (1.3%) to complete the procedure. More participants claimed they usually allow for 2 mm or 3 mm thickness of temporary restoration (39.8% and 32.8%, respectively) compared to 4 mm (24.2%), while only 3.2% selected 5 mm thickness.

The favorite temporary access cavity material was calcium sulfate-derived dental material such as Cavit (50.3%), followed by glass ionomer cement (GIC) (32%), interim restorative material (IRM) (13.5), and composite (2.9%). Around 1.3% of the participants mentioned the use of the double seal technique of Cavit and GIC or alternative brands of calcium sulfate-derived material such as Coltosol F. Cotton pellet was the favorite spacer material (88%) followed by Teflon tape (3.4%) and foam pellet (2.9%). While, 5.7% of the participants prefer not to use any spacer material.

Remarkably, 58.3% of participants claimed that they did not receive any courses or training in isolating and temporizing restoratively compromised teeth for endodontic procedures.

**Figure 3:**
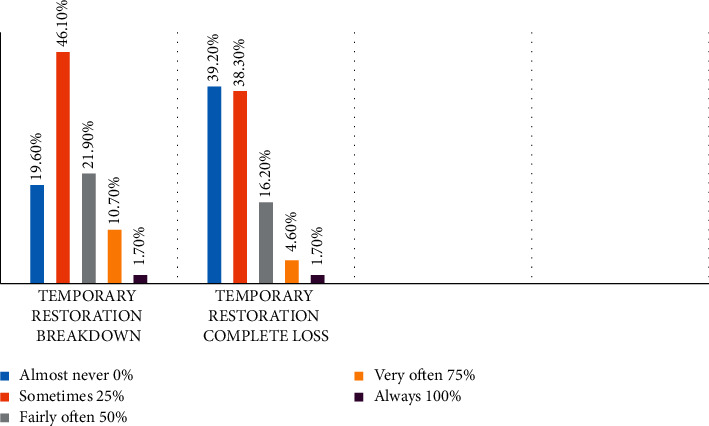
The frequency of clinically observing the deterioration or complete loss of access cavity temporary material in the second.

Moreover, 51.25% of endodontists were not exposed to special training or courses concerning this matter. Unfortunately, the breakdown or complete loss of temporary material was a common clinical observation as shown in Figure 3. Merely 19.6% and 39.2% of the participants, respectively, claimed that they rarely observed breakdown or complete loss of temporization correspondingly.

Practices in temporization were similar in the private and government sectors. Endodontists were more likely to perform single-visit RCT (*P* < 0.0001), use orthodontic or copper band to support interim restoration (*P*=0.04), use the double seal technique for temporization (*P* < 0.0001), and allow for greater thickness of temporary material (*P* < 0.0001) compared to other dental professionals in the study. Figure 2 shows the plot of nanoparticle size with respect to time, recorded over a 90 s period. The error bars represent the standard deviation of measurements for 20 particles in five separate sample runs (*n* = 100).

## 4. Discussion

The study showed that two-visit RCT was a common clinical procedure in Saudi Arabia. Unfortunately, the majority of participants noticed temporary restoration breakdown or complete loss in between RCT visits. Studying the clinician practices and trends in temporization will help to understand the causes of this frequent phenomenon. The calcium sulfate-derived material such as Cavit was the preferred temporization material followed by GIC and IRM. The duration between two RCT visits was two weeks or less, for 83.6% of the participants. Spacer placement was a common clinical practice in which 88% of the participants preferred to use cotton pellet. Similar preferences were observed when the diplomats of the American Board of Endodontists were surveyed for their temporization practices in 2006 [17]. They preferred to use Cavit as temporary material for anterior and posterior teeth, and 83% of the endodontists used cotton pellet beneath the restoration [17]. Calcium sulfate-derived materials were considered as an excellent temporary material when used in 4-5 mm thickness for less than 2-3 weeks [22, 23]. However, 72.6% of participants allowed for 2-3 mm thickness of temporary restoration. Although the duration between the two visits was less than two weeks for 83.6% of the participants, the smaller thickness of temporary restoration could be the potential cause of the observing temporary material breakdown or complete loss, especially when a spacer material such as a cotton pellet was used adjunctively which can act as a cushion and facilitate material breakdown under masticatory load [18, 19, 24, 25]. There are growing evidences that suggest Teflon is a better alternative spacer material [25, 26]. Jensen et al. and Abbott et al. [27] advised for complete removal of defective restoration before initiating to evaluate restorability of endodontically involved teeth [15, 26]. Also, advised for the placement of interim restoration such as GIC, composite, or amalgam to aid in isolation and support the function of the tooth [16]. Alternatively, the purpose of a temporary restoration is to cover the access cavity made within the interim restoration with a material that is easier to handle and remove such as Cavit [16]. Fortunately, more than 55% of the participants always remove the defective restoration before starting the RCT, and only 8.6% of the participants do not use an interim restoration to support temporary restoration in between RCT visits. Furthermore, in our study, applying the double seal technique was frequent, especially among endodontists, while the double seal technique was less practiced among the diplomats of the American Board of Endodontists [17]. The placement of stainless steel bands to support the interim restoration in teeth missing buccal or lingual walls was recommended in literature [16, 19, 20]. Most of the participants never used a band to support interim restoration which could further explain the frequent negative outcome of temporary material breakdown or loss. More than half of the participants claimed that they never received special courses or training in isolation and temporizing restoratively compromised teeth for endodontic procedures. The same was claimed by 51% of endodontists, although they were more likely to practice evidence-based techniques such as allowing for greater thickness of temporary restoration, using bands to support the interim restoration and the double seal technique in temporization. The American Association of Endodontists categorizes cases that require extensive pretreatment modification to improve isolation as cases of high difficulty that are even challenging for the most experienced clinician [28]. These skills may need to be taught with special attention in undergraduate and postgraduate dental programs. The study methodology is observational; therefore, limited information can be obtained regarding the cause of deterioration or complete loss of temporary restoration. Evidence-based practices in temporization were largely derived from in vitro microleakage studies [20, 22, 24, 29]. However, microleakage studies were regarded as clinically irrelevant, though they remain an important source of information for biomaterial comparison and helpful in clinical decision making [30, 31]. The scientific evidence for best practices in temporization can become subjective in the absence of enough support in the literature. The search for an ideal material for temporization is still needed because of frequent failure of available temporary material.

## 5. Conclusion

Two-visit RCT is commonly practiced in Saudi Arabia, and endodontists performed significantly more single-visit procedures. Temporization practices in Saudi Arabia may lack uniformity; however, clinicians were more likely to use calcium sulfate-derived material for two weeks or less. They allow for 2-3 mm restoration thickness and use a cotton pellet as a spacer. They completely remove the defective restoration before starting RCT and use interim restoration and the double seal technique with a variable degree of frequency. They rarely use bands to support interim restorations in restoratively compromised cases compared to the endodontist. The endodontist also significantly allowed for greater thickness of temporary restoration and used the double seal technique in temporization.

The clinical observation of temporary material breakdown or complete loss was frequent among the participants. This mandates further attention in undergraduate and postgraduate education to improve the clinical skills in isolating and temporizing restoratively compromised teeth. The need for further evidence to support the best clinical practices and materials in temporizing was emphasized to overcome the shortcoming in the current practices and available materials.

## Figures and Tables

**Table 1:** The survey instrument.

1. Where do you mainly do root canal treatment in your clinic time in Saudi Arabia? (a) Government. (b) Private. (c) None. (If the participant answered c, then the participant will be thanked and will exit the survey).
2. What is your last degree of qualification? If you are a resident, please select the option that matches your residency program. (a) general practitioner. (b) endodontist. (c) restorative dentist or advanced general dentist. (d) Intern/dental student.
3. How often do you practice these techniques in between two root canal treatment visits? (Five-points Likert format options: 0%, almost never; 25%, sometimes; 50%, fairly often; 75%, very often; 100%, always (100%) (a) Two visits root canal treatment. (b) Complete removal of previous defective permanent restorative material before RCT. (c) Place interim restoration (GIC or composite) to support temporary access material (Cavit). (d) Use an orthodontic band or copper band to support the interim restorative material. (e) The use of a spacer such as a cotton pellet or foam pellet in between two dental appointments. (f) The double seal technique using two restorative materials such as Cavit followed by GIC.
4. In the majority of your two visit cases, what is the duration between starting and completing RCT? (a) Two weeks or less. (b) One month. (c) One to three months. (d) More than three months.
5. What is the minimum thickness that you usually allow for your temporary access material? (a) At least 2 mm. (b) At least 3 mm. (c) At least 4 mm. (d) At least 5 mm.
6. What is your favorite access cavity temporary material? (a) Calcium sulfate-derived dental material such as Cavit or Cavit G. (b) Interim restorative material (IRM).

## Data Availability

The data used to support the findings of this study are available from Dr. Fahda Algahtani (fnalgahtani@pnu.edu.sa) upon request.
